# High-Quality Manufacturing with Electrochemical Jet Machining (ECJM) for Processing Applications: A Comprehensive Review, Challenges, and Future Opportunities

**DOI:** 10.3390/mi16070794

**Published:** 2025-07-07

**Authors:** Yong Huang, Yi Hu, Xincai Liu, Xin Wang, Siqi Wu, Hanqing Shi

**Affiliations:** 1 School of Power and Mechanical Engineering, Wuhan University, Wuhan 430072, China; 2 Hubei Key Laboratory of Waterjet Theory and New Technology, Wuhan University, Wuhan 430072, China; 3 Institute of Technological Sciences, Wuhan University, Wuhan 430072, China; 4 Center for Strategic Studies, Chinese Academy of Engineering, Beijing 100088, China

**Keywords:** electrochemical jet machining, non-traditional machining technology, micromachining, electrochemistry

## Abstract

The enduring manufacturing goals are increasingly shifting toward ultra-precision manufacturing and micro-nano fabrication, driven by the demand for sophisticated products. Unconventional machining processes such as electrochemical jet machining (ECJM), electrical discharge machining (EDM), electrochemical machining (ECM), abrasive water jet machining (AWJM), and laser beam machining (LBM) have been widely adopted as feasible alternatives to traditional methods, enabling the production of high-quality engineering components with specific characteristics. ECJM, a non-contact machining technology, employs electrodes on the nozzle and workpiece to establish an electrical circuit via the jet. As a prominent special machining technology, ECJM has demonstrated significant advantages, such as rapid, non-thermal, and stress-free machining capabilities, in past research. This review is dedicated to outline the research progress of ECJM, focusing on its fundamental concepts, material processing capabilities, technological advancements, and its variants (e.g., ultrasonic-, laser-, abrasive-, and magnetism-assisted ECJM) along with their applications. Special attention is given to the application of ECJM in the semiconductor and biomedical fields, where the demand for ultra-precision components is most pronounced. Furthermore, this review explores recent innovations in process optimization, significantly boosting machining efficiency and quality. This review not only provides a snapshot of the current status of ECJM technology, but also discusses the current challenges and possible future improvements of the technology.

## 1. Introduction

Electrochemical machining (ECM) has long been recognized as an effective technique for the precise machining of hard-to-machine materials. Among its various advancements, electrochemical jet machining (ECJM) has emerged as a powerful tool, offering unparalleled precision and versatility. ECJM utilizes a high-velocity electrolyte jet to remove material anodically, which allows for localized machining with high accuracy [1]. This method has found significant applications in fields requiring intricate and precise manufacturing, such as aerospace [2,3], biomedical [4,5], and microelectronics industries [6,7,8]. The development of ECJM has been driven by the need to achieve high-quality surface finishes and complex geometries that traditional ECM techniques struggle to produce. By focusing the electrolyte jet, ECJM enables localized material removal, minimizing the thermal and mechanical stresses typically associated with conventional machining processes.

Recent research in ECJM has concentrated on optimizing process parameters [7,9,10], improving electrolyte formulations [11,12], and developing advanced control systems to enhance precision and efficiency [13]. Innovations such as ultrasonic- [14,15], laser- [16,17], magnetism- [18,19,20], and abrasive-assisted ECJM [21,22,23] have further expanded the technique’s capabilities, allowing for finer control over material removal rates and feature dimensions. Additionally, the integration of real-time monitoring and feedback systems has enabled significant improvements in process stability and repeatability.

However, the majority of existing ECJM research has focused solely on the optimization of individual techniques and their associated parameters. Research that integrates multiple techniques to leverage their combined benefits remains limited. By rationally combining various techniques, it is possible to achieve synergistic performance improvements, thereby significantly enhancing the machining capabilities of ECJM. This review aims to provide a comprehensive overview of the current state of ECJM technology. It will cover the fundamental principles, recent advancements, its variant technology, and applications. Furthermore, the review will discuss the challenges and future directions in the field, emphasizing the potential for ECJM to revolutionize precision manufacturing. By consolidating recent findings and identifying key trends, this paper seeks to serve as a valuable resource for researchers and practitioners in the field of electrochemical jet machining.

## 2. Principle of Electrochemical Jet

**Figure 1:**
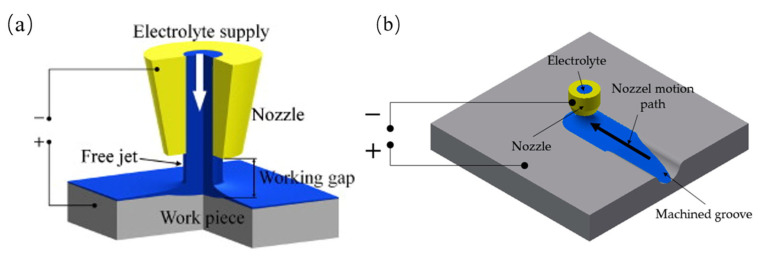
Schematic diagram of electrochemical jet principle: (**a**) 3D view of electrochemical jets; (**b**) scheme of ECJM.

In ECJM, a high-velocity jet of electrolyte is directed at the workpiece. A schematic diagram of the principle of ECJM is shown in Figure 1. The jet acts as a conductive medium, providing a localized area where the electrochemical reaction can occur. The electrolyte typically used is an aqueous solution containing ions that facilitate the anodic dissolution of the workpiece material, such as sodium nitrate (NaNO_3_) or sodium chloride (NaCl). The primary mechanism of material removal in ECJM is anodic dissolution. When the electric potential is applied, metal atoms from the workpiece lose electrons (oxidation) and form positively charged metal ions. These ions then dissolve into the electrolyte. For instance, for a metal *M*, the dissolution reaction can be represented as follows:

(1) $$M\to M^{n+}+ne^{-}$$

Here, $M^{n+}$ represents the metal ions formed, and $e^{-}$ denotes the electrons lost.

The dissolved metal ions are carried away from the machining zone by the flowing electrolyte jet. Effective mass transport is crucial to prevent the redeposition of metal ions on the workpiece and to maintain a consistent machining rate.

The calculable formula between the passed charge and the mass (*m*) of the removed material is expressed by Faraday’s law [24]:

(2) $$m=\left(\frac{Q}{F}\right)*\left(\frac{M}{Z}\right)$$

where *F* is Faraday’s constant (96,500 C/mol), *Q* is the total electric charge passed, *Z* is the valence number of ions of the anode (workpiece) material, and *M* is the molar mass of the material.

## 3. Electrochemical Jet Machining (ECJM)

Natsu and Kunieda [25] conducted two studies utilizing electrochemical jet machining (ECJM) to produce complex and three-dimensional surfaces. Their work builds on research from the 1990s, where Yoshida and Kunieda investigated the flow of electrolyte and the distribution of electric potential and current density on the workpiece surface [26]. They found that the current density followed a Gaussian distribution, concentrating within the machining area, which indicated the good localization of the ECJM process. Previous studies have applied ECJM technology to various metal-based and specialized materials [8,27]. Most experimental results have demonstrated the technique’s effectiveness and feasibility [28]. Lohrengel et al. [1] explored the application of electrochemical machining on hard alloys, particularly WC/Co materials. They investigated the electrochemical behavior of WC6Co and demonstrated the effectiveness of ECM using an alkaline electrolyte mixture. Using selected parameters, Liu et al. [27] efficiently machined “S”-shaped surface structures on Ti1023 titanium alloy by controlling the multidimensional nozzle movement. Further, Liu et al. [29] examined the application of jet electrochemical machining on TB6 titanium alloy. They identified optimal parameters of 24 V voltage, 0.6 mm inter-electrode gap, 2.1 L/min flow rate, and 15% sodium chloride electrolyte. Additionally, they studied the anodic behavior of TB6 titanium alloy in sodium chloride solution, proposing a novel simulation model based on anodic behavior to predict machining profiles and localized corrosion [30]. Hackert-Oschätzchen et al. [31] utilized ECJM for microstructuring hard alloys, achieving high-precision microstructuring on tungsten carbide alloys. Liu et al. [8] also investigated ECJM technology on SiC particle-reinforced aluminum matrix composites (SiCp/Al), analyzing the machining capabilities and material removal mechanisms. It was found that introducing abrasives into the ECJM process effectively removed the oxide layer on the aluminum surface, achieving higher machining rates than pure ECJM. The material removal mechanisms included matrix corrosion and SiC particle extraction, significantly impacting surface roughness due to these interactions.

**Figure 2:**
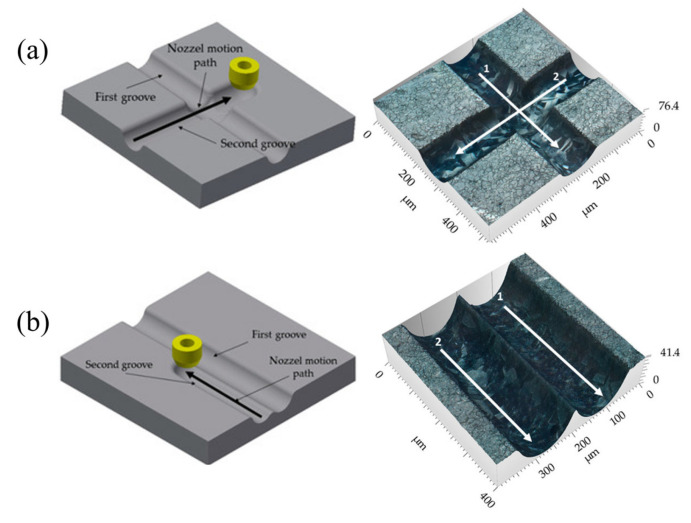
Experiment and results of grooves machining using a electrochemical jet with different machining paths; (**a**) intersecting; (**b**) parallel grooves [36].

To enhance ECJM technology, improvements in working parameters and mechanisms have become research focal points. Hackert-Oschätzchen et al. [32] studied the influence of jet shape on the machining process and how numerical simulations could predict and control this process. They developed a new model integrating fluid dynamics and the level set method for two-phase flow to simulate the dynamic formation of jet shapes. Wang et al. [33] investigated the impact of circular hydraulic jump (CHJ) on material removal characteristics in ECJM, establishing a multiphysics simulation model to calculate the overall shape, current density distribution, and workpiece deformation. They studied the effects of jet flow rate and the inter-electrode gap (IEG) on CHJ and material removal characteristics using experiments. Early models focused on coupling fluid dynamics and basic electrochemical reactions. However, for greater accuracy, it is important to account for the behavior of individual ions in the electrolyte, as their distribution significantly impacts current density and material removal. Wu et al. [34] employed mask electrolyte jet machining (MEJM) to develop a multi-ion model that thoroughly simulates ion diffusion, convection, and electromigration during the electrochemical machining process, elucidating the impact of ion concentration distribution on micro-cavity formation. The experimental outcomes robustly corroborated the model’s accuracy. Clare et al. [35] explored methods to enhance the precision of electrochemical jet technology through electro-physical modifications. Techniques such as altering jet angles, utilizing transient masking effects, and modulating energy density were studied for their impact on precision. Experimental results demonstrated the ability to achieve cut widths close to the nozzle diameter and quantified the individual contributions of energy density and length scales, showing that the feature resolution of electrochemical jet machining could rival other surface structuring techniques without thermal load. Yahyavi et al. [36] studied the role of online current density measurement in controlling stainless steel ECJM. As shown in Figure 2, they monitored current development during single-slot and cross-slot machining using systematic experiments to derive systemic impacts. The results showed that controlling current density allowed machining grooves with the desired depth and surface roughness, highlighting the sensitivity of current density to changes in the working gap and its applicability in process control through analysis and monitoring.

**Figure 3:**
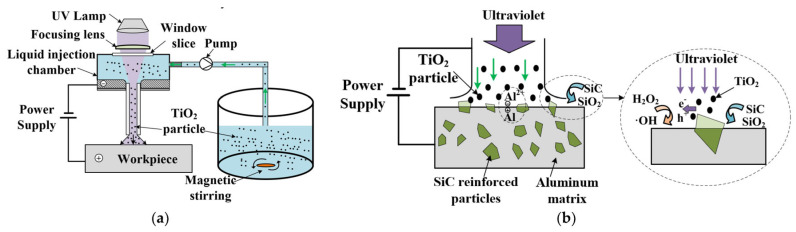
Photocatalytic–assisted jet electrochemical machining principle of composite SiCp/Al: (**a**) schematic diagram of PECJM; (**b**) processing principle [40].

**Figure 4:**
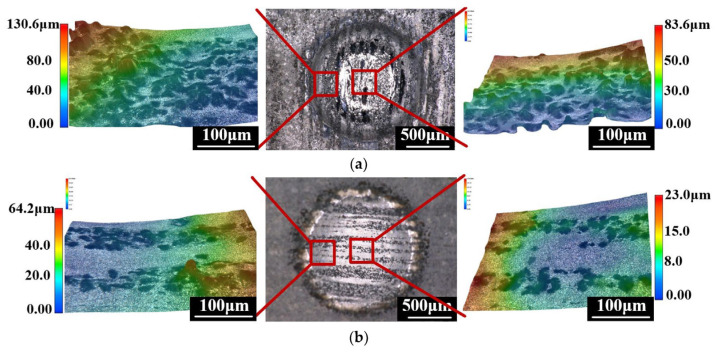
Comparison of microstructures at the processing voltage of 40 V: (**a**) JECM; (**b**) PAJECM [40].

To enable machining of new composite materials, Lu’s team introduced anodic discharge into ECJM, investigating its impact on semiconductor material 4H-SiC in electrochemical jet machining (ECJM) [37]. They explored the characteristics and control principles of anodic discharge behavior and how these factors significantly influenced material removal and EJM precision. An optimal surface finish (Ra 170 nm) and high shape accuracy were achieved by applying high-frequency AC (1000 Hz) without overcutting. To address challenges in microstructuring chemically inert and easily passivated materials, they proposed a new jet electrochemical plasma (Jet-EPM) microfabrication method [38]. This method intentionally introduced electrochemical plasma in the jet-material contact area to disrupt and remove the passivation layer, enabling material removal. Jet-EPM’s feasibility was validated using experiments on semiconductor material 4H-SiC, thoroughly studying its machining principles and characteristics. Additionally, the team explored jet electrochemical discharge machining (EDM) principles and methods for easily passivated metals like niobium [39]. They investigated overcoming plasma electrolytic oxidation (PEO) in the machining process to achieve effective material removal. The study found that discharge intensity and electrolyte chemistry were key factors. Using NaOH as the electrolyte resulted in higher removal efficiency (up to 1.92 mm/min) and better machining localization, while NaCl provided better surface quality (Sa 99.4 nm). Wang et al. [40] proposed a new hybrid processing method, photocatalysis-assisted jet electrochemical machining (PECJM), to improve machining capabilities by simultaneously removing the aluminum matrix and SiC particles. The mechanism and experimental setup of PECJM are shown in Figure 3. Experimental results indicated that PAJECM significantly reduced the protrusion height of SiC particles on the surface. As shown in Figure 4, compared to ECJM without photocatalysis, surface roughness values decreased and machining quality improved at the same processing voltage. The material removal mechanism was confirmed through electrochemical polarization curves and elemental analysis, establishing a qualitative model for PECJM’s material removal mechanism.

### 3.1. Electrolyte Type

In electrochemical jet machining (ECJM) technology, the choice of electrolyte significantly influences machining performance, including material removal rate, surface finish, and precision [41]. Commonly used electrolytes include NaNO_3_ (acidic) and NaCl (neutral). Different materials react distinctively with specific electrolytes. The selection of electrolyte is influenced by the material being machined to ensure effective electrochemical dissolution without adverse reactions like passivation (formation of a non-reactive surface layer). The dissolution rate of materials in the electrolyte affects machining speed and efficiency. For instance, sodium chloride (NaCl) and sodium nitrate (NaNO_3_) might be used for machining stainless steel, while other materials might require different electrolytes. Additionally, the electrolyte composition impacts the surface finish. More aggressive electrolytes might lead to faster material removal but could result in a rougher surface. Consequently, substantial research has been conducted on selecting suitable electrolytes for different materials.

**Figure 5:**
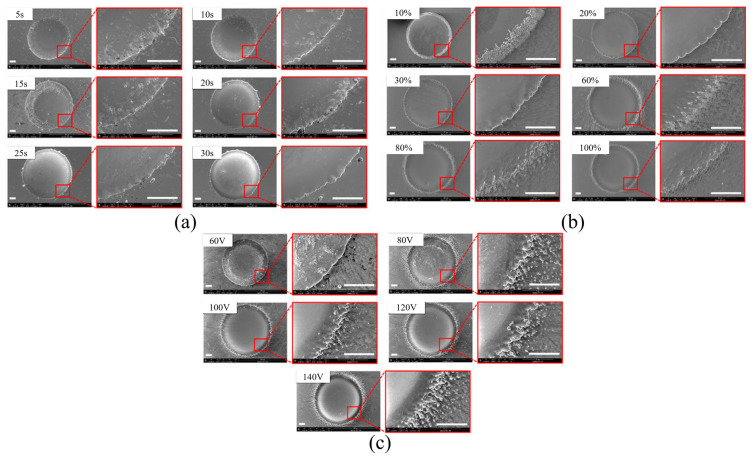
SEM images of micro-hole morphology of Zr-based bulk metallic glass processed using a NaCl–ethylene glycol electrolyte under different processing parameters: (**a**) different effective voltage times; (**b**) different duty cycles; (**c**) different voltages [42].

**Figure 6:**
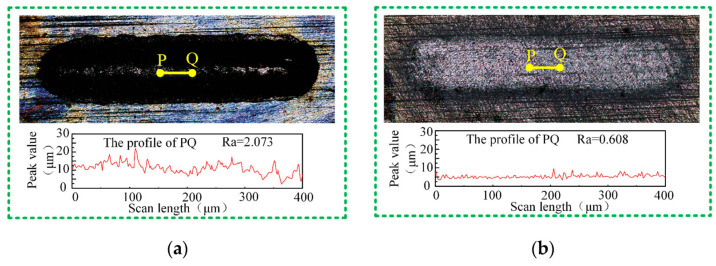
Grooves processed using (**a**) an aqueous NaCl electrolyte and (**b**) NaCl–EG electrolyte [12].

**Figure 7:**
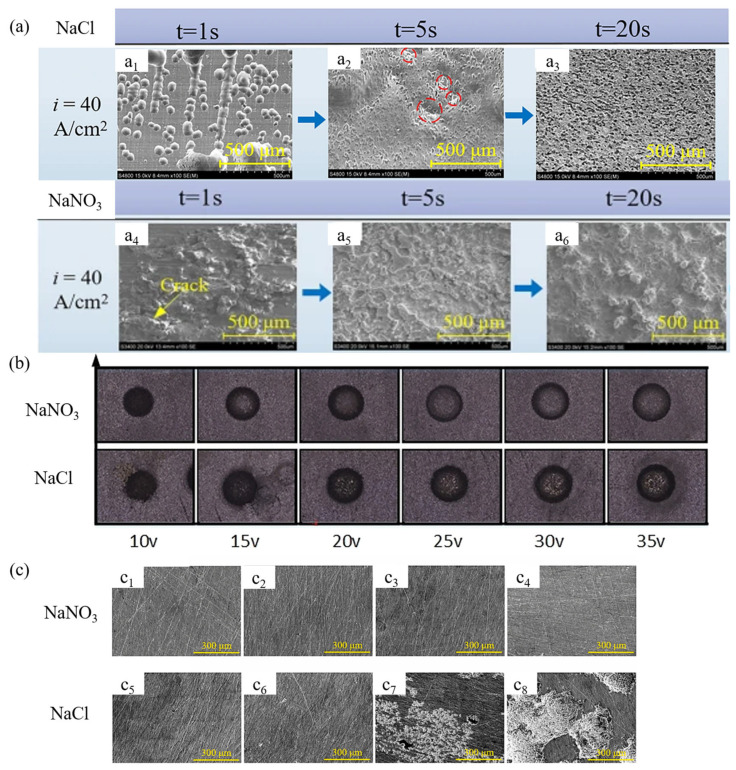
Comparison of SEM surface morphology of different metal materials processed with NaCl and NaNO_3_ as electrolyte solution: (**a**) titanium alloy; (**b**) SiC particle-reinforced aluminum matrix composites; (**c**) Zr702.

Guo et al. [42] investigated the dissolution mechanism and surface integrity of Zr-based BMGs (bulk metallic glasses) using a NaCl–ethylene glycol (EG) electrolyte. As shown in Figure 5, it was found that a NaCl–EG electrolyte enables uniform electrochemical dissolution of Zr-based BMGs, reducing the formation of oxide layers. Experimental results showed that using an alcohol-based electrolyte yielded desirable surface morphology, with micro-pit diameter and depth proportional to voltage and effective voltage time. Niu et al. [12] explored the use of NaCl–EG electrolyte in jet electrochemical micromachining (ECM) of Ti-6Al-4V titanium alloy. As shown in Figure 6, results indicated that ECM with NaCl–EG electrolyte significantly improved machining accuracy and surface quality. Ti-6Al-4V exhibited better corrosion resistance in NaCl–EG electrolyte, reducing stray corrosion. Figure 7 compares the SEM surface morphology of different metal materials processed by sodium chloride and sodium nitrate as electrolyte solutions. In these investigations, He et al. [43] studied the feasibility of using polyaluminum chloride (PAC) electrolyte in mask jet electrochemical machining (MJECM) to create micropore arrays on Zr702 alloy. The PAC electrolyte demonstrated a low self-corrosion potential and high passivation threshold potential during electrochemical corrosion of Zr702. Optimized parameters produced high-quality micropore arrays, including an average pore diameter of 152.11 μm, an average depth of 76.13 μm, and excellent dimensional consistency and surface quality. Liu et al. [44] examined the electrochemical dissolution behavior and jet electrochemical machining characteristics of TB6 titanium alloy in high-concentration salt solutions. Tests of current efficiency and polarization curves in various solutions revealed that electrochemical dissolution exhibited greater uniformity at high current densities (above 200 A/cm^2^), with significantly reduced stray corrosion. The surface quality of TB6 titanium alloy machined using 20 wt% NaCl solution was excellent, with a minimum surface roughness (Ra) of 0.373 μm. Subsequently, the team proposed a new hybrid method combining electrical discharge machining (EDM) and electrochemical jet machining (ECJM) for processing titanium alloys in highly conductive salt solutions [45]. This hybrid method not only improved material removal rate (MRR) but also significantly reduced erroneous corrosion effects, lowering surface roughness Ra from 0.800 µm to 0.177 µm. Liu et al. [27] also investigated the feasibility of jet electrochemical machining (ECJM) of TB6 titanium alloy, identifying the following optimal machining parameters: 24 V voltage, 0.6 mm electrode gap, 2.1 L/min flow rate, and 15% sodium chloride electrolyte. Additionally, they explored electrochemical jet machining (ECJM) of Ti1023 titanium alloy using NaCl–ethylene glycol-based electrolyte, examining the impact of oxide layer formation on machining accuracy [46]. A new electrolyte was proposed to overcome this issue, showing that NaCl–ethylene glycol-based electrolyte helps prevent oxide layer formation during ECJM, offering better overall performance compared to water-based electrolytes. The study also analyzed the effects of parameters when using a NaCl–ethylene glycol-based electrolyte, introducing the concept of high electrolyte temperature to achieve small-sized geometries in ECJM.

Lohrengel et al. [1] found that WC/Co alloys were challenging to achieve uniform electrochemical dissolution in neutral solutions (e.g., NaNO_3_ and NaCl). Introducing an alkaline electrolyte mixture (NaNO_3_ and NaOH) effectively facilitated ECJM of WC/Co. Improvements in electrolyte composition allowed successful electrochemical machining of WC6Co, reducing surface roughness and enhancing machining precision. Ao et al. [7] discovered that NaNO_3_ electrolyte is more suitable than NaCl for jet ECM of SiCp/Al, as it enhances machining accuracy. Results indicated that surface roughness depends on electrolyte choice and voltage, with NaNO_3_ electrolyte favoring smoother surfaces and higher voltage achieving greater depths. Optimized process parameters successfully produced well-shaped SiCp/Al grooves. To explore the application of novel electrolytes in titanium alloy electrochemical jet machining, Speidel et al. [47] compared the effects of using sodium halide (bromide, chloride, and fluoride) solutions with the commonly used sodium nitrate solution on surface finish, material removal rate, and pit formation. It was found that NaCl electrolyte, at concentrations below 2.5 M, increased the material removal rate by over 100% compared to sodium nitrate electrolyte. Additionally, doping NaCl electrolyte with sodium fluoride reduced overcutting effects in pits by half. Liu et al. [48] found that using pH-neutral NaCl solution for electrochemical slurry jet micromachining exhibited higher machining current density, corrosion rate, and machining accuracy compared to alkaline solutions. Further, they investigated the mechanisms of electrochemical jet machining (ECJM) of high-volume fraction SiCp/Al composites using NaCl electrolyte [6]. Experimental results showed that NaCl electrolyte offered better machining depth as machining time increased from 1 to 3 min, although positional accuracy was lower compared to using NaNO_3_.

### 3.2. Optimization of ECJM

In electrochemical jet machining (ECJM), stray corrosion refers to the phenomenon where the electrolyte, in a non-ideal flow state, affects surfaces outside the intended machining area, leading to corrosion [41,49]. This manifests as material dissolution or etching on parts of the workpiece or tool electrode beyond the desired machining zone. Such corrosion degrades the surface quality and potentially affects machining accuracy. Low current density regions, particularly at the edges of the workpiece and reflection areas of the jet, are prone to stray corrosion [50]. The main causes of stray corrosion include uneven electric field distribution, electrolyte flow state, and improper machining parameter settings. To minimize the detrimental effects of stray corrosion on machining quality, various measures and research have been proposed. Two representative approaches include modifying the shape of the electrochemical jet flow field (e.g., nozzle improvements [51,52], changes in jet form [53,54]) to mitigate stray corrosion effects and using masking methods to improve the localization of the electrochemical jet [55].

**Figure 8:**
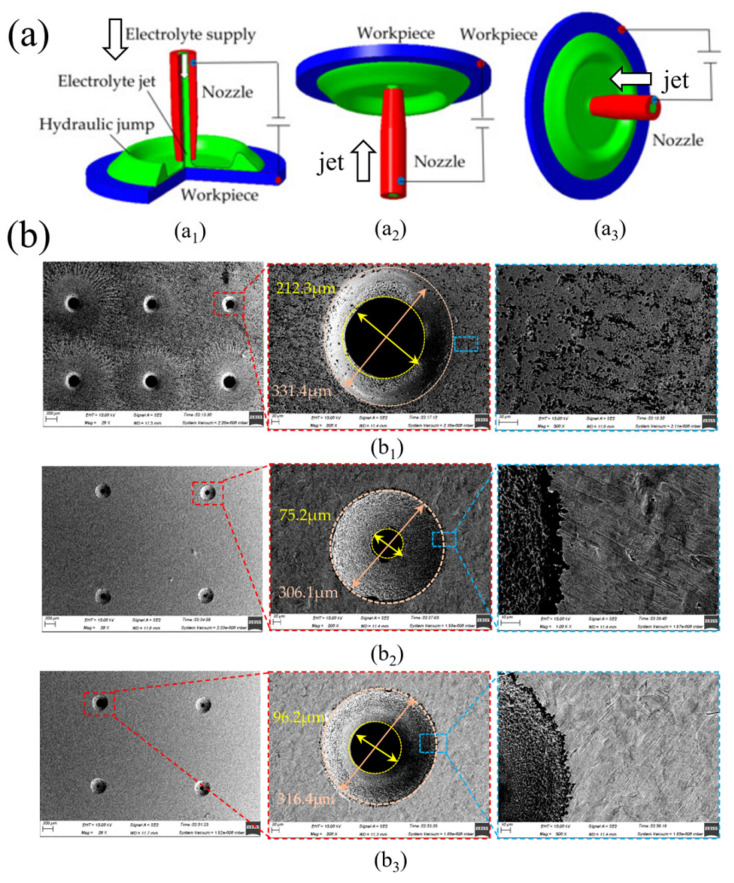
The influence of different jet orientation on the processing quality: (**a**) orientation diagram; (**b**) comparison of surface morphology; (**a_1_**,**b_1_**) vertically downstream mode; (**a_2_**,**b_2_**) vertically upstream mode; (**a_3_**,**b_3_**) horizontal mode [61].

Kong et al. [56] explored the use of ultra-high current density in ECJM to rapidly achieve the desired jet reflection shape, thereby reducing stray corrosion in titanium alloy processing. Simulations and experiments confirmed that ultra-high current density could quickly form the ideal jet reflection shape, effectively protecting unmachined surfaces from stray corrosion, thus enhancing machining efficiency and quality. Liu et al. [57] optimized the machining flow field in electrochemical turning (ECT) using numerical simulation to reduce gas mixing and improve the machining efficiency and surface quality of rotating titanium alloy components. Experiments demonstrated that the optimized flow field significantly improved machining efficiency and maintained the original profile shape, with a contour error of less than 1% and a surface roughness of approximately 2.414 μm. Kendall et al. [52] investigated the impact of nozzle geometry on material removal characteristics in ECJM, including nozzle designs with different diameters as well as convergent, divergent, and smooth features. It was found that the geometry of the nozzle bore significantly affected the machining profile, with convergent nozzles increasing cutting depth by up to 9.7% compared to symmetrical cylindrical channels. A 2D Star CCM+ simulation model was proposed, showing good agreement with experimental results. Wang et al. [58] proposed a novel cathode tool with a negative incident jet morphology to improve the product transport efficiency and machining performance in macro-ECJM of TC4 titanium alloy. Simulations and experiments indicated that using tools with negative incident jet morphology increased the high-speed flow region at the rear end and reduced low-speed flow regions in the machining area, thereby improving product transport efficiency and surface quality. Subsequently, to enhance the material removal rate in macro-ECJM of TC4 titanium alloy, the team proposed a tool design with a back-move jet channel [4]. Simulations and experiments revealed that the back-move jet channel significantly increased the charge at each point on the workpiece surface, thus improving MRR. When the back-move distance was increased to 1.5 mm, the MRR increased by 41%, the machining depth increased by 33%, and the taper angle decreased by 15%. Wang et al. [59] also investigated methods to reduce stray corrosion in ECJM by adjusting jet shapes. It was found that adjusting the jet shape significantly reduced stray corrosion at the machining edges, and the necessary conditions for completely eliminating stray corrosion were established. Zhao et al. [60] proposed a new surface modification method based on an electrochemical jet, called electrochemical jet hydrogenation (EJH), to selectively alter the brittleness of materials. This method was validated on niobium metal, achieving significant localized embrittlement, and the degree of embrittlement was precisely controlled by adjusting electrochemical parameters, primarily current density and processing time. Zhang et al. [61] studied the impact of jet orientation on the characteristics of ECJM, particularly on the fabrication of micro-sized features (Figure 8). It was found that horizontal jet orientation favored improved machining accuracy and surface quality, although the material removal rate was lower than that of vertical jet orientation.

Li’s team developed a novel high-precision kerosene-immersed horizontal ECJM technique, which impacts the workpiece horizontally rather than the traditional vertical direction [62]. Results showed that, compared to traditional vertical ECJM, horizontal ECJM achieved better machining localization, geometric consistency, and surface quality. The primary reason was that the horizontal jet significantly reduced the recontact of reflected electrolyte with the outer wall of the metal nozzle in use, thereby significantly reducing stray corrosion. This jet direction also helped remove electrolytic products from the machining area due to gravity effects, improving mass transfer conditions and stabilizing the machining process. Subsequently, they conducted an in-depth study of this technique, investigating the kerosene-immersed ECJM and the optimization of its electrochemical machining parameters to improve machining localization and accuracy [63]. It was found that the surface and shape effects of the workpiece significantly influenced machining localization and accuracy. Using kerosene medium instead of air medium improved ECJM’s machining localization. Compared to traditional ECJM, kerosene-immersed ECJM produced micro-sized features with higher precision and better surface quality on small surface planar and curved workpieces. The potential for fabricating fine features using jet-based electrochemical methods is significant. For instance, masked electrolyte jet machining (MEJM) has been used to create complex micro-protrusions and cavities with high precision in batch size [64], demonstrating the potential of this technique in efficiently producing precise surface microstructures in bulk [65], with specific applications including the fabrication of periodic features like micro-cavity arrays [66] and even micro-scale lettering [67]. Concurrently, other variants like mixed-gas EJM have also demonstrated advanced capabilities, such as the successful fabrication of micro-scale lettering [68]. Hung et al. [68] explored the application of ECJM in coating workpieces with and without mixed gas and electrolyte. Particularly, electrochemical machining experiments with and without gas mixing were conducted to produce microscale blind holes and grooves, comparing the efficiency of both processes. It was found that mixed gas and electrolyte improved machining efficiency and reduced the conductive area between the electrolyte and the workpiece, thereby enhancing current density on the workpiece surface and increasing material removal rate. Low voltage could lead to insufficient current density, causing stray corrosion on the material, affecting machining characteristics and surface roughness. In slot ECJM, increasing the electrode feed rate could reduce machining time per unit area, excessive cutting depth and lateral cutting, and improve surface roughness.

## 4. Electrochemical Jet Composite Processing Technologies

### 4.1. Ultrasonic-Assisted ECJM

In traditional electrochemical jet machining (ECJM), the chemical reactions near the electrode generate by-products such as gas bubbles [26]. As these by-products accumulate, they degrade machining stability and surface quality. Additionally, a passivation oxide layer forms on the workpiece surface, preventing effective electrochemical reactions between the electrolyte and the fresh metal substrate by forming an insulating layer, thereby obstructing the desired material removal [28]. These two main issues result in low accuracy, poor surface quality, and reduced stability in conventional ECJM.

**Figure 9:**
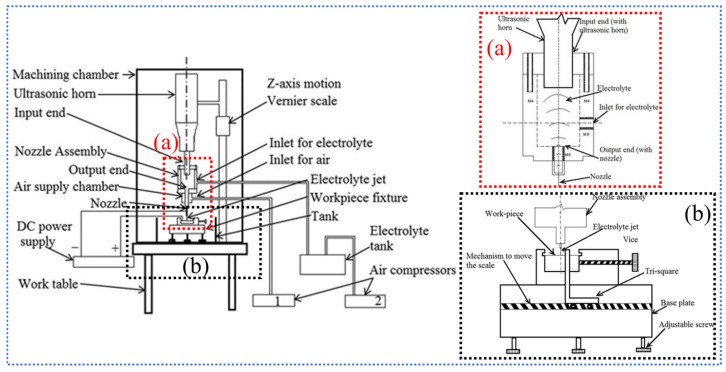
Typical schematic diagram of an ultrasonic-assisted electrochemical jet machining device. (**a**) The schematic diagram of nozzle working principle; (**b**) The diagram of workpiece processing.

To enhance the machining quality and efficiency, researchers have proposed the use of ultrasonic-assisted electrochemical jet machining (UECJM) [69]. UECJM is an advanced micromachining technology that combines the principles of electrochemical machining (ECM) with the benefits of ultrasonic vibration assistance [15]. Typically, UECJM achieves high-frequency vibration of the electrolyte by coupling an ultrasonic transducer with a nozzle. Within the nozzle cavity, the ultrasonic vibrations of the transducer end face create alternating pressures, causing the electrolyte to form a jet as it exits the nozzle. The pulsed jet impacts the target material, forming an electrochemical circuit with the applied power source. The schematic diagram of the typical ultrasonic assisted electrochemical jet processing device is shown in Figure 9.

The assistance of ultrasonic vibration in UECJM can be categorized into three primary effects: (i) High-Frequency Vibration: Ultrasonic vibrations generate a pulsed jet, where the alternating fluid pressure significantly enhances the water hammer effect, increasing the impact force on the target [70]. This improvement enhances the material removal capability, facilitates the removal of the passivation oxide layer, and improves machining stability. (ii) Ultrasonic Cavitation: The addition of ultrasonic vibration induces cavitation in the electrolyte [3]. Microbubbles in the electrolyte undergo growth, expansion, and collapse under the influence of acoustic pressure. During cavitation, the collapse of these microbubbles produces high-speed micro-jets (with speeds up to 350 m/s [71]) that effectively remove the oxide layer on the workpiece surface, thereby improving the efficiency of the electrochemical process. (iii) Enhanced Electrolyte Flow: Ultrasonic vibration improves the flow properties of the electrolyte, enhancing the removal of reaction by-products and improving the contact between the electrolyte and the workpiece surface [72]. This enhancement increases machining accuracy and surface quality.

Singh et al. [73] first proposed using ultrasonic-assisted electrochemical honing (UECH) to improve the machining accuracy and surface quality of bevel gears. By applying ultrasonic vibrations to the workpiece surface, the performance of the electrochemical honing (ECH) process is enhanced. Experimental results indicate that ultrasonic vibrations act as performance multipliers in ECH, facilitating the easy and complete removal of oxide layers from the ECM-processed workpiece surface. The use of ultrasonic significantly improves the average and maximum surface roughness of bevel gears, achieving up to 91.04% and 71.98% improvement, respectively, with an optimal ultrasonic frequency of 42 kHz. Skoczypiec et al. [74] hypothesized that ultrasonic vibrations influence the electrochemical dissolution process through cavitation phenomena and applied a full cavitation model to simulate this effect. Numerical studies demonstrated that ultrasonic vibrations alter the conditions of electrolyte flow and electrochemical dissolution within the gap, leading to increased machining allowance and removal rates, which depend on the amplitude of the ultrasonic vibrations. The impact of ultrasonic vibrations on the ECM process includes improved removal of heat and reaction products in the machining area, support for diffusion, reduced passivation rates, altered electrochemical machining coefficients, and the creation of optimal hydrodynamic conditions from the perspective of the surface layer. Goel et al. [72,75,76,77] conducted several studies on ultrasonic-assisted electrochemical jet micro-drilling. They developed an experimental setup for producing micro-holes in copper workpieces using the electrochemical drilling process, including pure jet electrochemical micro-drilling, gas-assisted, ultrasonic-assisted, and pulsed direct current-assisted jet electrochemical micro-drilling. The effects of parameters such as voltage, electrolyte concentration, inter-electrode gap, and electrolyte pressure on process responses, including hole taper and material removal rate (MRR), were investigated. Experimental results indicated that ultrasonic vibrations significantly affect both MRR and hole taper, with MRR notably increasing and hole taper decreasing compared to pure jet electrochemical micro-drilling. To further explore ultrasonic-assisted jet electrochemical micro-drilling, an apparatus was constructed that included a specialized nozzle assembly for transmitting ultrasonic vibrations in the electrolyte jet and a workpiece fixture. Results showed that combining ultrasonic vibrations with ECJM significantly enhanced micro-drilling performance. The optimal conditions, including a pulse-on time of 1.2 s, a voltage of 50 V, an inter-electrode gap of 3.25 mm, an electrolyte concentration of 1.25 M/L, and an electrolyte pressure of 0.12 MPa, resulted in a minimum hole taper of 0.38 degrees. Goel et al. [72] also utilized a multiphysics coupling modeling approach to simulate the flow pattern of the electrolyte jet and the material removal process, comparing the simulation results with previous experimental outcomes. This comparison validated the accuracy of the model and the effectiveness of ultrasonic assistance in improving MRR. The study explored how ultrasonic assistance enhances fluid movement in the electrolyte via acoustic energy, improves electrode kinetics, and removes the passivation layer in electrochemical machining. The effects of ultrasonic assistance on pressure fields, current density, and workpiece material reaction rates were also discussed.

Besekar et al. [78] introduced a novel vibration-assisted axial nozzle jet wire electrochemical machining (WECM) technique to improve the machining precision and quality of metal micro-components. Compared to conventional axial flow WECM, the new technique achieved a 36% reduction in slit width and a 75% improvement in machining accuracy. The vibration-assisted axial nozzle jet WECM technique showed excellent performance in enhancing machining accuracy and reducing slit width, with potential applications in micro-slit machining of NiTinol shape memory alloy (SMA). The team further investigated the wire electrochemical machining (WECM) process of NiTinol SMA using a low-concentration sulfuric acid (0.1 M H_2_SO_4_) aqueous acidic electrolyte [79]. The effects of parameters such as electrolyte flow rate, tool piezoelectric ceramic (PZT) amplitude and frequency, and electrolyte temperature on machining characteristics were studied. Using multiple experiments, a homogeneous minimum slit width of 110 microns with a standard deviation of 0.57 microns was achieved under controlled machining conditions. Wang et al. [80] studied the application of ultrasonic vibration technology in electrochemical micro-drilling and how using a double-layer insulation (TiO_2_ ceramic coating and organic film) enhances the durability of electrode sidewalls. Experiments demonstrated that the double-layer insulation exhibited strong durability in ultrasonic-assisted electrochemical micro-drilling. Machining accuracy stabilized when the workpiece vibration amplitude exceeded a certain threshold. The proposed electrode sidewall insulation method significantly improved the durability of the insulation layer in ultrasonic-assisted electrochemical micro-drilling, contributing to enhanced machining accuracy. Tong et al. [71] investigated the material removal mechanisms, particularly cavitation corrosion, of 6061 aluminum alloy in ultrasonic-assisted electrochemical micromachining (USEMM). Through simulations and experiments, it was found that the speed of micro-jets could reach 350 m/s, resulting in the formation of plastic micro-pits on the metal surface. Electrochemical polarization behavior indicated that during USEMM, a passivation layer formed and was rapidly disrupted on the metal surface. The micro-jets generated by ultrasonic cavitation significantly influenced material removal, leading to the formation of uniform and flat pits, which improved surface quality compared to conventional electrochemical micromachining (EMM). Chen et al. [81] prepared high-performance Ni-Co-WC nanocomposite coatings using ultrasonic-assisted scanning jet electrodeposition and studied the effect of WC nanoparticle concentration in the electrolyte on coating performance. Experimental results showed that ultrasonic vibrations improved the surface morphology, mechanical properties, and corrosion resistance of the coatings, with hardness increased by 15.9%, adhesion strength increased by 76.1%, and corrosion current density reduced by 13.0%. The Ni-Co-WC nanocomposite coating, prepared with a WC concentration of 6 g/L, exhibited optimal surface quality and performance, significantly enhancing the protection and service life of marine platform support structures.

**Figure 10:**
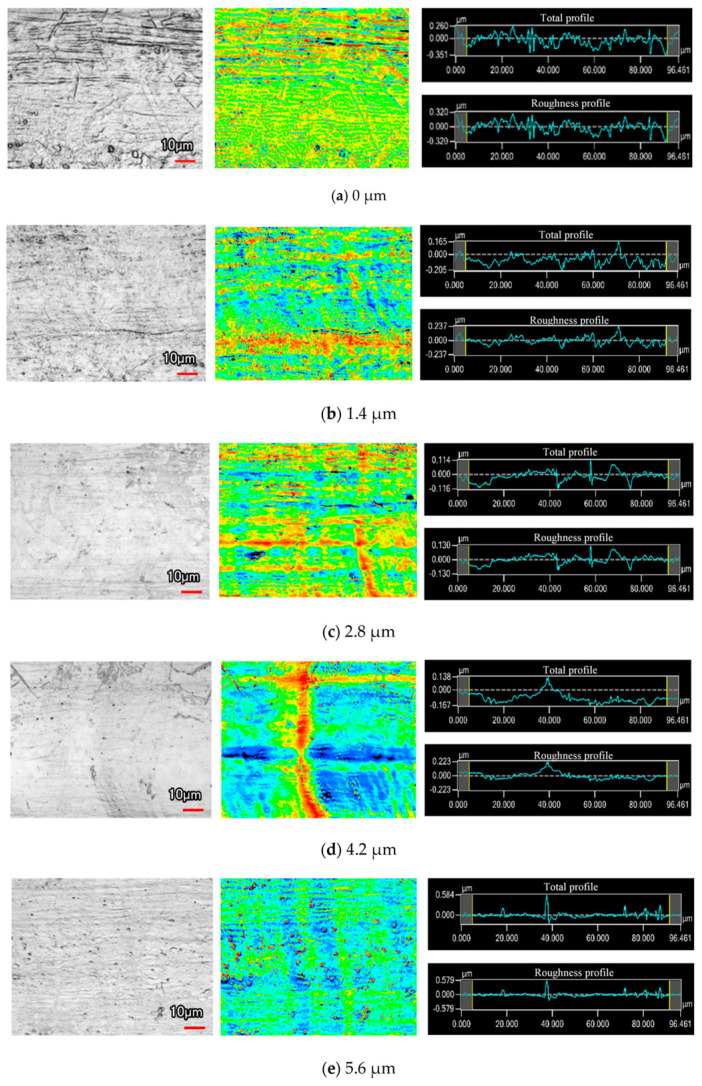
Surface morphology under different vibration amplitudes [87].

Yang et al. [82] proposed an electrochemical cutting method assisted by workpiece vibration in the feed direction, using a tubular electrode with inclined jet holes. Results showed that workpiece vibration-assisted electrochemical cutting effectively improved machining efficiency and precision, successfully creating array slice structures on stainless steel blocks. Workpiece vibration accelerated electrolyte renewal and removal of electrochemical products, enhancing machining efficiency and precision. At a vibration amplitude of 0.1 mm and frequency of 1.5 Hz, the average feed rate increased by 50%, and the width difference between the upper and lower ends of the slit decreased from 115.56 μm to 49.6 μm. Mitchell-Smith et al. [83] reported at a conference on the use of ultrasonic-assisted electrochemical jet machining (UECJM) technology to overcome the formation of passivation layers on titanium alloys during electrochemical machining. Experimental results confirmed that ultrasonic assistance could partially remove the passivation layer, improving machining performance, increasing depth, minimizing kerf width, and reducing surface roughness Ra along the groove bottom by 31%. Wang et al. [84] utilized ultrasonic-assisted disk micro-tools for electrochemical micro-hole machining (EMM) in stainless steel to create deep micro-holes. By optimizing parameters, high aspect ratio micro-holes were successfully fabricated, demonstrating the potential of this method for creating deep microstructures in metal materials. Results indicated that disk micro-tools could concentrate the electric field, enhancing localized dissolution. Ultrasonic vibrations significantly improved machining speed and maximum hole depth while reducing hole taper and diameter, thus improving surface quality. Subsequently, their team investigated the impact of ultrasonic cavitation micro-jet impacts on material corrosion in ultrasonic-assisted electrochemical micromachining (UAEMM) [85]. The impact pressure of micro-jets was calculated, and simulations validated the effect on material corrosion pits, revealing that material removal was more pronounced in protruding areas. Ultrasonic vibrations were found to significantly enhance the material removal rate and surface precision, increase surface hardness, and improve the performance of electrochemical micromachining. Li et al. [86] proposed a micro-rotary ultrasonic-assisted electrochemical drilling (UA-ECD) method to improve machining accuracy, efficiency, and stability. Numerical simulations and mathematical models were used to study the effects of ultrasonic vibrations and key processing parameters on machining outcomes. The UA-ECD method was found to significantly enhance machining quality, efficiency, and stability. Optimized comparative experiments successfully produced high-quality micro-hole arrays on 304 stainless steel workpieces, demonstrating the substantial potential and wide application prospects of the rotary ultrasonic-assisted micro-electrochemical drilling method. Ling et al. [87] introduced a novel ultrasonic-assisted wire electrochemical micromachining (UA-WECMM) method to improve WECMM performance. The surface morphology of the target material under different vibration amplitude processing is shown in Figure 10. Flow field simulations and experimental validations showed that ultrasonic vibrations significantly increased mass transfer efficiency in the machining gap, reduced slit width, and improved surface morphology. Optimized parameters enabled the successful fabrication of T-shaped micro-connectors and micro-gears with good surface roughness on stainless steel workpieces of various thicknesses, demonstrating the potential and application prospects of the UA-WECMM method. Dalabehera et al. [88] experimentally analyzed electrochemical jet machining of thin metal sheets under ultrasonic vibrations with continuous and pulsed direct current. It was found that the application of pulsed direct current and ultrasonic vibrations was suitable for precision machining, with ultrasonic vibrations facilitating tighter dimensional control at higher frequencies. While pulsed direct current was suitable for precision machining under all conditions, ultrasonic vibrations provided advantages in dimensional control for fine feature generation at higher frequencies. Che et al. [3] proposed a novel ultrasonic-assisted electrochemical polishing method for NiTi alloys and constructed the corresponding equipment. Using the Box–Behnken experimental design method, the effects of ultrasonic amplitude, voltage, and temperature on surface roughness were investigated. It was found that surface quality of NiTi alloys improved after ultrasonic-assisted electrochemical polishing. This technology, by combining ultrasonic energy fields, expanded the application range of electrochemical polishing and provided a new method and foundation for polishing NiTi alloys with complex geometries in additive manufacturing.

### 4.2. Laser-Assisted ECJM

**Figure 11:**
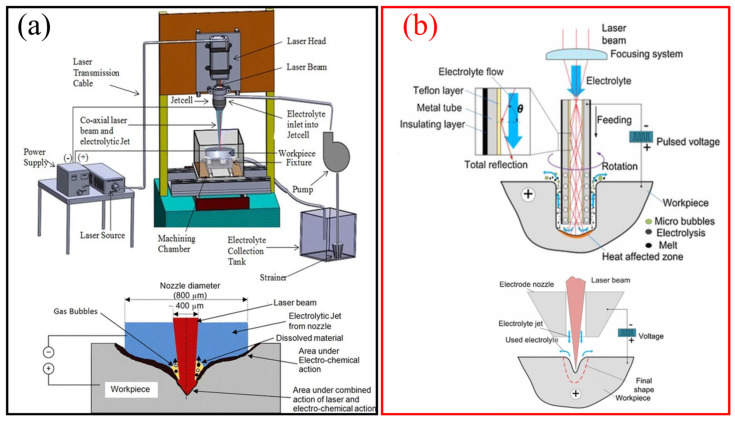
Schematic diagram of two methods of laser-assisted ECJM: (**a**) coaxial parallel coupling; (**b**) based on internal total reflection coupling.

Laser-assisted electrochemical jet machining (LAECJM) represents an advanced hybrid machining process that combines the strengths of laser processing and electrochemical machining (ECJM). This technology leverages the precision and controlled energy delivery of laser beams with the electrochemical dissolution process to achieve high-precision, complex geometries in difficult-to-machine materials. The process integrates a focused laser beam with an electrochemical jet. The laser locally heats the workpiece, enhancing the electrochemical reaction rate by increasing the conductivity and reactivity of the material surface [17]. This dual action significantly improves material removal rates and machining precision. The processing mechanism of laser-assisted ECJM mainly involves the following: (i) Laser Interaction: The laser beam, typically from a Nd or fiber laser, is directed onto the workpiece, creating localized thermal effects. This heating softens the material and may induce localized melting, reducing the energy required for electrochemical dissolution [89]. (ii) Electrochemical Jet: An electrolyte jet, composed of a conductive solution (e.g., NaCl or NaNO_3_), is directed toward the heated area. The jet acts as an electrode, with the workpiece serving as the counter electrode. When a voltage is applied, an electrochemical reaction occurs, selectively removing material from the workpiece surface [16]. (iii) Synergy of Processes: The combination of thermal softening from the laser and the electrochemical reaction accelerates material removal, allowing for finer control over the machining process. This synergy reduces mechanical stresses and thermal damage, common in traditional machining processes [90]. At the same time, the target material absorbs the laser energy to change the temperature, which strengthens the surface current density distribution and contributes to the electrochemical reaction. Laser ablation can further remove the oxide layer formed through anodic dissolution and subsequent oxidation processes to ensure stable processing [91]. Figure 11 shows two kinds of laser-assisted ECJM coupling methods and processing schematics. One is parallel coupling, and the other is light-based internal total reflection coupling.

In 2004, P.T. Pajak and colleagues [92] attempted to combine low-power laser machining technology with electrolyte jet machining to enhance the precision and quality of electrochemical jet machining. Their experimental research focused on the precision and efficiency of laser-assisted jet electrochemical machining (LAECJM). The results indicated that LAECJM demonstrated superior machining efficiency and precision in micro-hole machining compared to single jet electrochemical machining. Mathematical modeling and experimental analyses supported these findings. Subsequent studies by Pajak et al. [93] revealed that LAECJM technology significantly improved machining precision and productivity, with a notable increase in material removal rates for various metal materials. Additionally, the shape and positional accuracy, as well as the surface roughness of holes and cavities, were enhanced. To further investigate the mechanisms of this technology, A.K.M. DeSilva and colleagues [94,95] examined the thermal effects in LAECJM. Their experiments and finite element modeling revealed that laser assistance could significantly increase the material removal rate while maintaining the machining area’s temperature within a safe range, thereby preventing electrolyte boiling and heat-affected zone damage. Zhang et al. [96] studied a novel hybrid process—jet electrochemical machining assisted by laser drilling (ECJM-LD)—aimed at minimizing recast layers and spattering defects during laser drilling. Experimental results showed that, in the ECJM-LD process, recast layers and spattering were effectively reduced compared to laser drilling in the atmosphere. They measured laser attenuation in the electrolyte, finding that green laser attenuation was primarily due to scattering, whereas infrared laser attenuation was due to absorption. The team validated the hybrid process’s effectiveness in reducing recast layers and spattering using further experiments and proposed a two-dimensional mathematical model describing the shape of holes processed by ECJM-LD [97]. By optimizing laser parameters and environmental conditions, they achieved high-precision hole machining [98]. In their latest research [99], they applied this technology to 0.5 mm thick 321 stainless steel, achieving a reduction of over 90% in recast layers and over 95% in spattering. They simulated and experimentally validated the temperature and electric fields during laser drilling and jet electrochemical machining. Yuan et al.’s [100] experimental results similarly indicated that using an electrolyte jet to guide the laser to the workpiece for drilling, along with electrochemical dissolution, could effectively reduce spattering and recast layers. They optimized the composite process, particularly by determining the optimal distance between the anode and the workpiece to enhance efficiency and precision. Long et al. [101] conducted experimental research on laser-enhanced electrochemical micromachining (LIECM) of stainless steel, exploring the composite process combining laser beams and electrolytes. The results demonstrated the feasibility of a mask-free, single-step method for pattern transfer, although the etching rate was low. The etching rate was significantly increased using a jet-supplied electrolyte and a smaller cathode-anode gap. Malik and his team [102] studied precision micro-hole machining using a method that combined pulsed laser and jet electrochemical machining, developing a novel laser-assisted jet electrochemical machining (LAECJM) system. The results showed that LAECJM, when machining Inconel-718, reduced taper by 32.3%, overcut by 27.8%, and the average spark-affected zone by 50.2% compared to jet electrochemical machining. They optimized the LAECJM parameters using the Taguchi method to improve machining precision. Further findings indicated that LAECJM improved material removal rates by 29.7% compared to traditional JECM, while laser assistance enhanced machining quality and reduced processing time [91]. To optimize this technology further, the team employed multi-response optimization of LAECJM parameters based on grey relational analysis, using the Taguchi method for experimental design [103]. They studied the micro-drilling performance of LAECJM on Inconel-718, identifying the optimal parameter combination to achieve maximum material removal rate, minimum taper angle, and surface roughness.

Currently, research primarily focuses on two technologies: nozzle-based laser electrochemical machining and laser beam chemical etching. These techniques have shown promising results in surface treatment of small parts. However, nozzle-based laser machining has limitations in terms of precision and achievable aspect ratio. To address issues in machining accuracy, depth limitations, and taper angle control in existing laser and electrochemical machining (LECM) processes, Wang et al. [104] proposed a novel LECM process based on internal total reflection (ITR). In LECM-ITR, the electrolyte jet and laser beam are synchronously guided and transmitted to the machining area. Experimental results showed that LECM-ITR reduced side gap by 23.6%, reduced taper angle by 60.8%, and increased material removal rate by 118.8%. The mechanisms by which LECM-ITR improves electrochemical machining efficiency and precision were discussed, including laser-induced temperature elevation in the machining area increasing current density, and the formation of a thicker anodic oxide layer enhancing machining precision. In subsequent research [104], the team proposed a hybrid machining process combining the advantages of electrochemical machining (ECM) and laser beam machining (LBM) known as synchronous laser and tubular electrochemical machining (Laser-STEM). The mechanisms of Laser-STEM were studied using theoretical and experimental analyses, and a mathematical model was developed to investigate the impact of laser-induced thermal effects on electrochemical dissolution rates. Using the Laser-STEM process, under appropriate laser power, side gap was reduced by 62.7% and feed rate increased by 108% compared to non-laser-assisted machining. Experimental results indicated that machining efficiency increased with higher laser power, pulse voltage, and feed rate, while precision was enhanced with higher laser power and feed rate and lower pulse voltage. Ultimately, a diameter of 1.25 mm without a recast layer was successfully machined in 5 mm thick aluminum alloy workpieces. To further optimize the application of this technology, improvements were made to the Laser-STEM process based on previous research for machining deeper and more precise holes. Experimental results demonstrated that laser assistance increased current density in the machining area, thereby enhancing electrochemical machining removal rates. Using a custom-developed experimental setup, micro-cavities 2 mm deep and holes 5 mm deep were successfully fabricated [105]. Comparisons of different machining parameters showed improvements in precision and material removal rates by 60.7% and 122.7%, respectively. It was also found that higher laser power improved machining precision and material removal rates. Saxena’s team conducted research on tool-based laser-electrochemical micromachining processes, proposing a novel tool-based laser-electrochemical micromachining method [106]. This method utilized the synergistic action of laser and electrochemical processes along the same machining axis, enhancing the potential of both techniques while compensating for and minimizing their respective limitations. Experimental results indicated that laser assistance improved material removal rates and, within a specific processing window, produced high-quality surfaces with fewer defects. Metallographic studies of the machined surfaces revealed multiple removal mechanisms, such as laser removal, laser-assisted electrochemical removal, and electrochemical removal, depending on the applied laser pulse energy. Subsequent optical ray tracing simulations, multi-physics electrochemical micromachining process simulations, and experimental validations confirmed the feasibility of the hybrid tool concept [107]. However, due to initial design limitations, only 30–40% of the laser power could be utilized in the machining area.

Zhu et al. [108] proposed a new method for laser and electrochemical jet machining, presenting a self-coupling technique combining picosecond laser irradiation with electrochemical dissolution for efficient localized machining of germanium. By creating localized high-temperature regions on the germanium surface, the technique increased free electron density and conductivity, forming a highly localized conductive channel. The theoretical mechanism was experimentally validated, and the machined surfaces were thoroughly characterized. It was found that under picosecond laser irradiation, current increased by 3 to 5 times, reaching up to 225 mA, enabling the formation of smooth-surfaced, steep-walled pits at the bottom of germanium wafers in neutral and non-corrosive NaNO_3_ electrolyte. Additionally, a method for creating oxide-free pit structures on germanium surfaces using electrochemical jet machining combined with opposed laser irradiation was explored [109]. Using a NaNO_3_ electrolyte jet generated by a cathode nozzle, the germanium wafer bottom was vertically impacted, while a nanosecond laser irradiated from the opposite side to precisely enhance local conductivity. This method effectively removed residual oxide particles common in electrochemical machining, achieving oxide-free pit structures. Fluid field simulations of the electrolyte revealed velocity and pressure distribution patterns, deepening the understanding of the formation mechanisms of complex surface morphologies. This method successfully manufactured oxide-free pit structures with potential industrial applications.

Speidel et al. [110] investigated the surface modification of low-carbon steel using a combination of laser and electrochemical processes. Using laser pretreatment followed by electrochemical jet machining (ECJM), changes in surface roughness and hardness were studied. Experimental results showed that combining laser pretreatment with ECJM altered the exposed surface texture and chemical composition. The treated surface roughness (Sa) increased from approximately 0.45 μm to about 18 μm, and hardness increased from approximately 261 HV to over 700 HV. Surface chemical analysis revealed increased oxide formation on the pretreated ECJM surface, contributing to the increased surface roughness. Duan et al. [111] utilized electrochemical corrosion-assisted laser drilling to remove recast layers from metal micro-holes. An electrochemical machining head based on a tungsten wire electrode was designed, and the impact of auxiliary gases on the quality of electrochemical corrosion (ECC) treatment during laser drilling was studied. It was reported that oxidative auxiliary gases (such as compressed air and oxygen) weakened the removal rate of recast layers during ECC due to the formation of insulating oxide layers on the hole walls during the thermal chemical reactions of laser drilling. Non-oxidative auxiliary gases (such as nitrogen or argon) effectively removed recast layers. The proposed method improved efficiency by more than four times compared to traditional electrochemical machining techniques.

### 4.3. Magnetism-Assisted ECJM

**Figure 12:**
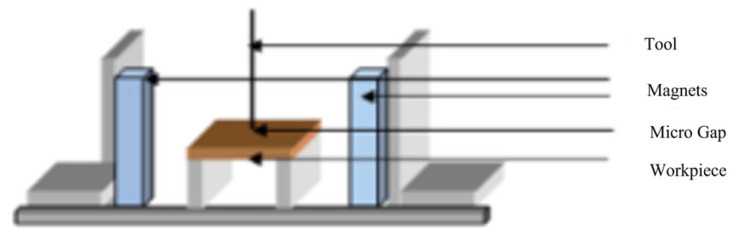
A schematic diagram of a magnetic-assisted ECJM processing device.

When a magnetic field is applied perpendicular to the direction of ionic current in the electrolyte, it induces a magnetohydrodynamic (MHD) effect (see Figure 12). This effect generates additional fluid motion within the electrolyte, which can enhance the transport of ions and reaction products away from the machining area [112]. Improved mass transport results in more efficient removal of dissolved material, reducing the risk of localized overheating or concentration polarization, both of which can adversely affect machining precision and surface quality. The introduction of a magnetic field can also induce turbulence in the electrolyte flow. Turbulent flow increases the rate of mixing and reduces the boundary layer thickness at the electrode surface, further enhancing ion transport. This results in more uniform material removal and a smoother surface finish [113].

**Figure 13:**
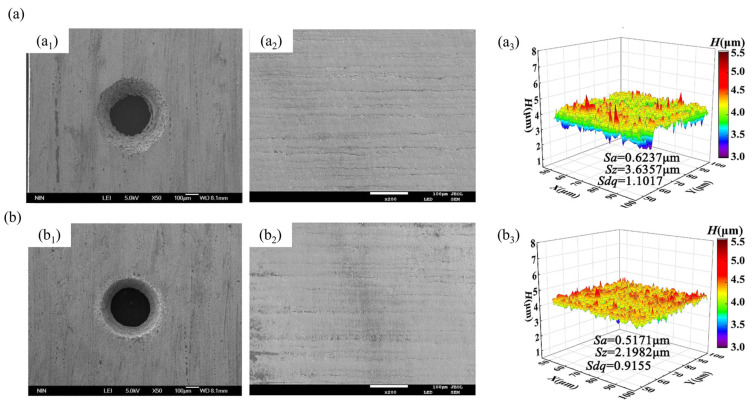
Comparison of machining quality of micro-holes and surfaces processed by ECJM and magnetic-assisted ECJM: (**a**) ECJM; (**b**) magnetism-assisted ECJM.

Wang et al. [19] investigated the impact of magnetic field-assisted electrochemical jet micromachining (MECJM) on the surface precision of 201 stainless steel. Using experiments and simulations, it was found that magnetic field-assisted ECM significantly reduced surface roughness and improved machining efficiency by accelerating charge and mass transfer in the electrolyte via magnetohydrodynamic convection. The team further explored the mechanisms by which this technique enhanced surface quality and suppressed stray corrosion [18]. Experimental results indicated that magnetic field-assisted ECM achieved an optimal surface morphology with a roughness of approximately 0.30 μm within 10 min, significantly reducing surface roughness and effectively suppressing stray corrosion. In their latest research [20], the team examined the effect of magnetic field-assisted electrochemical machining on the surface morphology of 2Cr13 stainless steel. Results demonstrated that ECM effectively reduced surface roughness, and the introduction of a magnetic field further enhanced machining efficiency. Simulations revealed that the magnetic field increased current density and accelerated anodic dissolution. Lin et al. [114] explored the effects of magnetic field and ethanol addition on electrochemical discharge machining (ECDM) of quartz glass. Experimental results showed that ethanol stabilized the square wave power supply waveform, reduced the contact angle between the electrode and bubbles, and improved machining stability. Increasing the magnetic field and voltage frequency reduced film thickness, enhanced hole roundness, and minimized taper. Pang et al. [115] analyzed the influence of locally induced magnetic field deformation on electrochemical micromachining behavior. It was found that the introduction of a magnetic field promoted mass transfer and accelerated anodic dissolution through magnetohydrodynamic convection. However, using dual magnetic poles arranged with the same polarity weakened this effect. Zhai et al. [116] proposed a rotating magnetic field-assisted electrochemical machining method to improve the surface roughness of multi-step internal taper holes. By designing a new cathode structure embedded with NdFeB permanent magnets on its sidewalls, the influence of the magnetic field on the uniformity of the machining gap flow field was simulated. Experimental results showed that the rotating magnetic field significantly improved workpiece surface quality and surface roughness. Li et al. [117] studied the effect of a magnetic field on anodic dissolution in electrochemical machining. Using COMSOL 5.2 software, an electrochemical reaction model was established to investigate the influence of magnetic field strength and direction on the electrolyte mass transfer rate and anodic dissolution. Simulation results indicated that the magnetic field enhanced the mass transfer rate and anodic dissolution, with the magnetic field direction being more critical than its strength. Experimental results were consistent with the simulations. Figure 13 shows the machining quality comparison between ECJM and magnetically assisted ECJM for micro-holes and surfaces.

Liao et al. [118] examined the effect of a magnetic field on the electrochemical dissolution behavior and surface quality of Ti-48Al-2Cr-2Nb alloy in electrochemical machining. Results showed that the magnetic field increased the response rate of adsorption reactions, enhanced decomposition potential and sensitivity to localized corrosion, suppressed current density fluctuations, and reduced the dissolution rate during the electrochemical corrosion process. The magnetic field positively affected corrosion uniformity, contributing to isotropic micro-surface quality. Zhang et al. [119] conducted an in-depth study on the impact of a magnetic field on the precision of micro-hole electrochemical drilling. It was found that an external magnetic field perpendicular to the feed direction improved machining precision, with the optimal machining precision achieved at a magnetic flux density of approximately 0.1 T. This research aids in enhancing micro-electrochemical drilling precision and advancing magnetic field-assisted micro-ECM technology. To better understand the mechanisms of magnetic field addition in electrochemical machining, Ophelia Frotscher et al. [120] investigated the Lorentz force-induced flow of NaNO_3_ electrolyte in magnetic field-assisted electrochemical machining. The study aimed to understand the process better and lay the foundation for subsequent process simulations using experiments and simulations. The experimental setup was designed with a magnetic field perpendicular to the electric field, and particle image velocimetry, magnetohydrodynamic, and multiphase flow simulations were used to study the flow. T. Praveena Gopinath et al. [2] studied the effect of a magnetic field on the electrochemical micromachining (ECMM) process of Ti-6Al-4V titanium alloy. They found that the magnetic field improved machining precision and performance characteristics through magnetohydrodynamic (MHD) effects. Experimental results showed significant improvements in the material removal rate (MRR) and overcut (OC) under attracting and repelling magnetic fields, with surface roughness reduced by 55.34% and no introduction of machining-related stress.

### 4.4. Abrasive-Assisted ECJM

**Figure 14:**
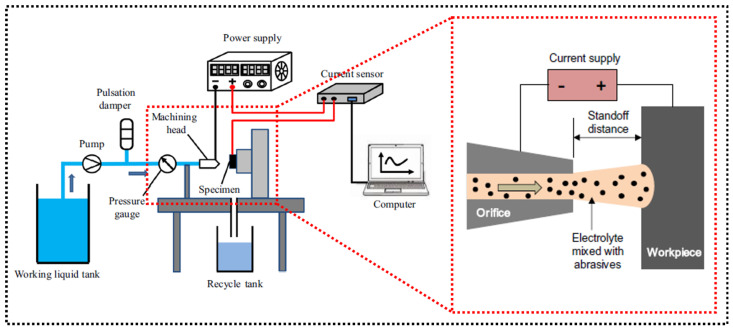
Schematic diagram of the abrasive-assisted ECJM machining device.

Electrochemical jet machining (ECJM) is a non-traditional machining process known for its precision in machining conductive materials without physical contact, thereby avoiding tool wear and thermal damage. However, the relatively slow material removal rate limits its industrial applications. Abrasive-assisted electrochemical jet machining (AECJM) enhances the ECJM process by integrating abrasive particles into the electrolyte jet, which increases the material removal rate and surface quality [121]. It combines two primary mechanisms: electrochemical dissolution and abrasive erosion. In AECJM, the workpiece acts as the anode, and the jet serves as the cathode. When a voltage is applied, the electrolyte facilitates the removal of material through anodic dissolution. The electrochemical process is responsible for dissolving the workpiece material at the targeted machining site. Moreover, abrasive particles such as silicon carbide (SiC) or aluminum oxide (Al_2_O_3_) are suspended in the electrolyte jet. These particles strike the workpiece surface at high velocity, providing mechanical erosion that aids in the material removal process [122]. Additionally, the abrasive action is particularly beneficial in removing the passivating oxide film that can form on the surface of the workpiece during electrochemical machining. This oxide film typically inhibits further dissolution, but its removal by abrasives allows the electrochemical process to continue more efficiently [6]. This dual-action approach increases the overall material removal rate and allows for more precise and efficient machining. The advantages of AECJM can be summarized as follows: (i) Increased Material Removal Rate: The combined action of electrochemical dissolution and abrasive erosion significantly enhances the material removal rate. (ii) Improved Surface Quality: The dual mechanisms provide a superior surface finish compared to conventional methods. (iii) Capability to Machine Hard Materials: AECJM is effective in machining hard and brittle materials, including superalloys, ceramics, and composites. (iv) Minimal Tool Wear: The non-contact nature of electrochemical machining reduces tool wear and extends tool life. Figure 14 is a schematic diagram of the abrasive-assisted ECJM processing device.

**Figure 15:**
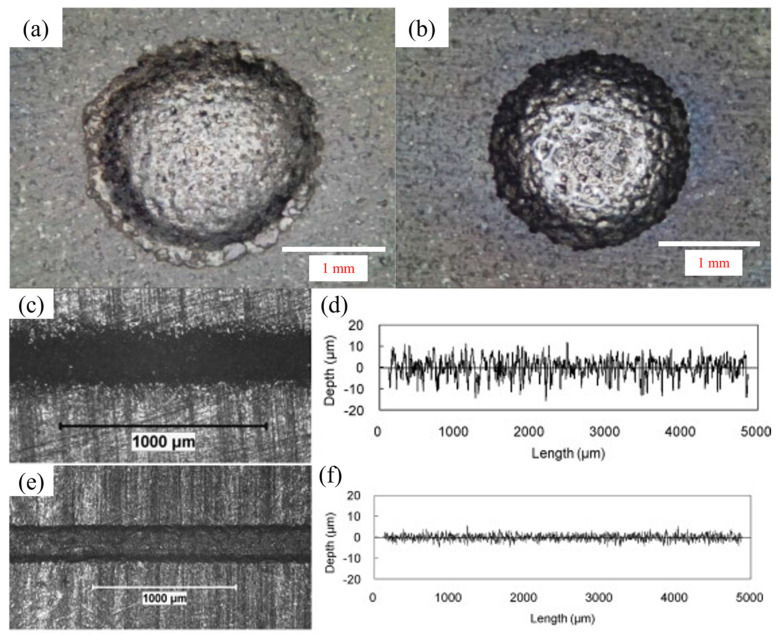
Comparison of surface morphology of micro-pits and micro-grooves processed using ECJM and AECJM: (**a**) stereogram for ECJM—no added abrasive particles; (**b**) stereogram for ECJM—using #2000 SiC abrasive particles; (**c**) photograph and (**d**) optical profilometer scan of centerline of channel machined using ECJM with 2.0 MPa jet pressure; (**e**) photograph and (**f**) optical profilometer scan of micro-channel machined using ASJM with 0.5 wt% Al_2_O_3_, 0.05 mm/s scan speed, 2.5 mm standoff, and 2.0 MPa jet pressure.

Through extensive research and technological advancements, significant progress has been made in the field of abrasive-assisted electrochemical jet machining (AECJM) technology. Table 1 summarizes recent studies on AECJM, including the power sources, specimen materials, and abrasive types. It is evident that Al_2_O_3_ and SiC are predominantly used abrasives, conventional metal-based materials are the primary target materials, and acidic electrolytes are commonly employed. The surface morphologies of micro-pits and micro-grooves processed by ECJM and AECJM are shown in Figure 15. Initially, Liu et al. [123] innovatively proposed using AECJM for micromachining metals, which integrates abrasive slurry jet machining (ASJM) and electrochemical jet machining (ECJM). Stellite 12 alloy was used as the target material in experiments, comparing the mass loss rate and surface roughness of samples processed by ASJM, ECJM, and AECJM under varying abrasive concentrations and jet pressures. The results confirmed that AECJM surpasses ASJM and ECJM alone in material removal rate and surface quality. Positive and negative synergistic effects were observed, with the latter occurring when the total mass loss rate exceeded approximately 0.4. Subsequent research by Liu and his team further explored AECJM technology. They applied AECJM to various metal materials to verify its feasibility, including tungsten carbide (WC) [48], SS316 stainless steel [122], 304 stainless steel [23], and high-volume fraction SiCp/Al composites [8]. The results demonstrated the high efficiency and economic viability of AECJM for these target materials. The effects of working voltage, abrasive concentration, jet velocity, and solution concentration on AECJM processing of WC were analyzed. The mechanism was identified as the removal of the oxide layer to expose uncorroded WC, achieving a higher current density and corrosion rate as well as localized machining compared to using ECJM alone. Additionally, the characteristics of micro-channel machining on 304 stainless steel using AECJM were investigated. An empirical model was proposed to predict the width and depth of the micro-channels, and experimental results validated the model’s reliability, with an overall deviation controlled between 4.4% and 6.3%. Zhao et al. [124] also developed a mathematical model to accurately predict the surface roughness area (Sa) of micro-channels fabricated on SS304 metal using AECJM. Unlike Liu et al.’s model, this model considered six major factors (working voltage, abrasive concentration, electrolyte concentration, jet pressure, jet scanning speed, and pass number) and their two-factor interactions. Validation showed that predicted values closely matched experimental data, with a maximum error of 12.2% and an average error of 3.5%. Despite its higher error, this model is reliable due to its consideration of more influencing factors. In the study of the machining capabilities and material removal mechanisms of ECJM and AECJM for high-volume fraction SiCp/Al composites, it was found that the removal mechanisms include matrix corrosion and the extraction of SiC particles. The presence of abrasives in AECJM facilitates the rapid removal of the passive oxide film formed by electrochemical corrosion, leading to a higher machining rate. To enhance the efficiency of AECJM for SiCp/Al composites, the use of a larger stand-off distance (SOD) was investigated to mitigate localization issues. Experimental results from Ref. [22] showed that a larger SOD reduces stray corrosion of the matrix material during the initial machining stage and prevents secondary dissolution due to recontact between the nozzle and reflected jet in subsequent stages.

In addition, Yehia et al. [125] conducted experimental studies on the effects of adding Al_2_O_3_ powder to electrochemical grinding on the metal removal rate and surface roughness. Experiments were performed on K110 alloy steel under varying voltages, feed rates, electrolyte concentrations, and cutting depths. The results indicated that adding Al_2_O_3_ improves the metal removal rate and reduces surface roughness, with optimal results achieved at a 5 wt% concentration. Other than conventional metals, AECJM has also been applied to novel alloys and metal composites. Tsai et al. [121] investigated the surface machining effects of AECJM on Ti-6Al-4V alloy. Their experiments revealed that a 0.05 wt% abrasive flow effectively removes the TiO_2_ oxide film on Ti-6Al-4V alloy surfaces. Under a processing pressure of 0.4 MPa and a gap of 0.4 mm, a machining efficiency of 20 mm/s was achieved. As AECJM technology has matured, Zhang et al. [5] proposed a method for the efficient precision machining of Inconel 718. A comparative analysis was conducted on three different machining methods: abrasive jet machining (AJM), electrochemical jet machining (ECJM), and AECJM. Energy-dispersive spectroscopy (EDS) was used to analyze the machined areas, assess removal efficiency, and microstructural characteristics [126,127]. It was found that the removal rate of AECJM significantly exceeded the combined rates of AJM and ECJM. The primary removal mechanism was electrochemical corrosion, with abrasive erosion playing a supporting role [5]. The microstructure of Inconel 718 significantly influenced the removal mechanism of AECJM.

Abrasive-assisted electrochemical jet machining represents a significant advancement in the field of precision machining, offering enhanced material removal rates and superior surface quality. Its ability to machine hard and complex materials makes it an invaluable tool in various high-tech industries. Continued research and development are expected to address current challenges and unlock the full potential of this hybrid machining technology.

**Table 1:** Summary of abrasives and working parameters used in the study.

References	Abrasive Type	Specimen	Voltage
[123]	Al_2_O_3_	Stellite 12 alloys	30 V
[23]	Al_2_O_3_	Stainless steel 304	-
[48]	-	WC alloys	60 V, 80 V, 100 V, 120 V
[125]	Al_2_O_3_	K110 alloy steel	9 V, 11.5 V
[22]	Al_2_O_3_	SiCp/Al composites	160 V
[122]	Al_2_O_3_	Stainless steel 316	80 V, 120 V, 160 V
[8]	-	SiCp/Al composites	150 V
[121]	SiC	Ti-6Al-4V alloys	60 V
[124]	Al_2_O_3_	Stainless steel 316	-
[5]	SiC	Superalloy Inconel 718	200 V

## 5. Challenges and Possible Improvements in Each Technologies

Electrochemical jet machining (ECJM) has emerged as a vital technology for processing complex geometries and fine features in hard-to-machine materials. Its variants further enhance its capabilities. This paper systematically reviews the principles, current research status, and progress of these technologies. Briefly, the advantages of various assisted techniques include the following: UECJM: Increases processing speed and surface finish. LAECJM: Enhances precision in hard materials. MECJM: Improves material removal efficiency and uniformity. AECJM: Achieves higher removal rates and better surface finishes for tough materials. However, several issues and challenges hinder the widespread adoption and efficiency of these technologies. This paper aims to explore these aspects and propose future research directions:

1. Most studies focus on ECM of materials like stainless steel and titanium alloys. However, experimental research on other advanced materials (e.g., superalloys, shape memory alloys, metamaterials, and high-entropy alloys) and various metal–matrix composites is significantly less reported in the literature. These studies are often conducted through a few selected random trials or using a single variable method. Future research should focus on the applicability and advantages of ECJM for these new alloys and composites. Artificial intelligence and machine learning can be utilized for real-time monitoring, enhancing processing precision and reducing human intervention through adaptive control algorithms. For example, data-driven ECM models using machine learning have shown promise in profile prediction and process understanding [128,129,130].

2. Although some research has been conducted on the impact of electrolyte jet shape on processing quality, the quantity is limited. The key to electrochemical jet machining lies in maintaining a stable processing electric field for efficient anode material removal. Variations in jet stability lead to inconsistent processing results, affecting dimensional accuracy and surface finish. Ensuring a stable and precise jet, maintaining a uniform electric field, and achieving even material removal remain challenges for the technology’s further development. Precise and stable jets can also minimize stray corrosion, resulting in higher surface quality. Future research should consider using gas and masks to shape the jet and optimize operational parameters to enhance jet stability, potentially through advanced mask designs or specialized nozzle configurations like duckbill nozzles and tilt nozzles [34,131,132].

3. The choice of electrolyte is crucial for electrochemical machining technology. The electrochemical behavior between the electrolyte and the target material varies for different anode materials. In past reviews, sodium nitrate solution has been the most commonly used electrolyte. Although some novel electrolytes (e.g., alcohol-based [41,133,134] and passivation electrolytes [135,136,137,138,139]) have been proposed and studied, other new electrolytes are needed to enhance the electrochemical corrosion efficiency of the anode during electrochemical jet machining. Especially for new material processing, further research is needed to understand the electrochemical behavior of proposed efficient electrolytes. Moreover, the waste electrolyte poses environmental threats, making waste treatment and disposal critical for the green industrialization of ECJM. Developing biodegradable and recyclable electrolytes and adopting closed-loop systems for electrolyte recovery and reuse will reduce costs and environmental impact, becoming a focus of this technology.

4. Ultrasonic-assisted electrochemical jet machining (UECJM) integrates ultrasonic vibration, electrochemical dissolution, and high-speed jetting for advanced material processing [140]. The mechanical vibration of ultrasonic enhances the electrochemical reaction efficiency while the high-speed fluid dynamics of the jet remove material, achieving high precision and efficiency [69,141,142]. The advantages include improved processing accuracy and surface quality as well as reduced thermal impact and mechanical stress, making it suitable for hard and difficult-to-machine materials. However, it faces challenges like equipment complexity, precise control, and balancing material removal rates. Existing research does not sufficiently address the synchronization of ultrasonic vibrations with electrochemical reactions to avoid interference with electrolyte flow, which significantly reduces jet stability. Furthermore, the local thermal effects caused by ultrasonic vibrations have not been adequately considered. Future research should develop AI- and machine learning-based real-time monitoring and control systems to optimize the synchronization of ultrasonic vibrations with electrochemical reactions. Establishing comprehensive simulation models incorporating fluid dynamics, electrochemistry, ultrasonics, and thermal effects will be effective in elucidating the mechanisms of UECJM and promoting technological innovations.

5. Laser-assisted electrochemical jet machining (LAECJM) uses the high-energy density of laser beams to locally heat the material surface, combined with the precise etching of electrochemical processes and dynamic material removal by jets, to achieve efficient and precise material processing. This technology provides extremely high processing precision and surface finish, suitable for various materials, including metals, ceramics, and semiconductors. Utilizing the non-contact nature of lasers reduces mechanical stress and offers flexibility for machining complex three-dimensional structures. Challenges include effectively controlling laser parameters to minimize thermal effects on materials, enhancing processing precision and efficiency [143], optimizing the synergy between laser and electrochemical reactions [17], and reducing equipment complexity and cost. Future research directions include developing intelligent laser control systems, exploring new electrolytes to reduce thermal effects [144], combining multi-physics field simulations to optimize processing parameters [145], and studying wear-resistant coatings and materials to improve equipment durability, thereby advancing LAECJM in high-precision machining fields.

6. Magnetic field-assisted electrochemical jet machining (MECJM) regulates the electrochemical reaction by applying a magnetic field, enhancing material dissolution rates while using jets to clear the machining area, achieving efficient and uniform material removal. The advantages include reduced processing time, uniform machining effects, minimized redeposition phenomena, and suitability for various materials. However, the key to improving material removal efficiency uniformly is the addition of a homogeneous magnetic field in the electrochemical jet machining environment. The current literature lacks sufficient research on this scientific issue, and the underlying mechanisms have not been fully explored. Future research should investigate the mechanisms of magnetic field effects on electrochemical processes, optimizing processing parameters through various methods to achieve the best machining quality.

7. Abrasive-assisted electrochemical jet machining (AECJM) involves introducing abrasive particles into the high-speed jet during electrochemical dissolution, achieving efficient material removal. This method enhances material removal rates, processing precision, and surface finish, particularly for hard and difficult-to-machine materials. Current research mainly focuses on premixed abrasive jets, with limited studies on post-mixed jets. While the introduction of abrasives can improve the erosive capability and material removal performance of the jet, the impact on the jet’s electric field distribution remains unknown. The distribution of abrasives within the jet flow field significantly affects the jet’s impact force concentration. Analysis of used abrasives indicates that alumina is commonly used. Beyond conventional parameter optimization, introducing new abrasives will be crucial for technological advancement. The embedding of abrasives into the target material after impact, which affects surface finish, is another overlooked area that needs improvement.

8. To overcome the limitations of electrochemical jet machining, scholars have proposed various assisted techniques, such as ultrasonic fields, magnetic fields, lasers, and abrasives. However, research on these techniques is conducted independently. The synergistic assistance of these technologies has not been explored. Future research can utilize decision-making methodologies (e.g., Grey relational theory [146,147], utility theory [148], Taguchi method [113,149,150]) and metaheuristic optimization algorithms (e.g., ant bee colony [151], genetic algorithms [152]) for multi-response optimization. Comparative analysis of these optimization outputs may assist future researchers in selecting more suitable optimization methods.

By addressing the bottlenecks and challenges of ECJM and its variants through targeted research and technological advancements, their capabilities and applications in precision manufacturing will be significantly enhanced. Focusing on advanced control systems, sustainable practices, hybrid methods, material advancements, and simulation models can unlock the full potential of these technologies.
